# Double-seedlings and embryo-free seeds generated by genetic engineering

**DOI:** 10.3389/fpls.2022.999031

**Published:** 2022-10-03

**Authors:** Yumei Xia, Yao Wang, Yuanyi Hu, Yijie Zhan, Junhao Dan, Ning Tang, Junyou Tian, Mengliang Cao

**Affiliations:** ^1^ State Key Laboratory of Hybrid Rice, Hunan Hybrid Rice Research Center, Changsha, China; ^2^ National Center of Technology Innovation for Saline-Alkali Tolerant Rice in Sanya, Sanya, China; ^3^ Long Ping Branch, Graduate School of Hunan University, Changsha, China

**Keywords:** apomixis, polyembryony, embryo-free seeds, haploid, endosperm development

## Abstract

Apomixis can fix the heterosis of Hybrid F_1_, by maintaining its heterozygous genotype, and is an ideal way for the development of hybrid rice. In this paper, we designed an engineering strategy for realizing apomictic reproduction of hybrid rice in the way of induce adventitious embryos. An embryogenesis gene, *AtWUS*, controlled by the ovule-specific promoter, a ribonuclease gene *Barnase* driven by the egg cell-specific promoter *pDD45*, and an inactivation gene *ZmAA1* driven by the pollen-specific promoter *pG47* were simultaneously integrated into one T-DNA, and co-transformed with the second T-DNA carrying a *Barstar* gene. Double-seedlings were observed in transgenic line. Whole-genome sequencing and ploidy levels confirmed by flow cytometry showed that one of the double-seedlings was heterozygous diploid and the other seedling was homozygous haploid, which confirmed that embryogenesis in one of the double-seedlings arises from the zygote after fertilization and the other derived from an unfertilized gamete. Meanwhile we obtained embryo-free seeds at frequencies of 2.6% to 3.8% in T_1_ generation, and 0.75% to 3% in T_2_ generation. Though we did not obtained adventitious embryos in hybrid rice in this study, the phenomenon of double-seedlings and embryo-free seeds in transgenic line was informative and strongly suggested that endosperm development is an autonomously organized process in rice, independent of egg cell fertilization and embryo-endosperm communication. This provides novel insights into the induction of haploid embryos and lends theoretical support to successful clonal propagation using synthetic apomixis

## Introduction

The wide commercial application of ‘three-line’ and ‘two-line’ hybrid rice has helped to guarantee the absolute grain security for 1.4 billion people in China (Ren et al., 2016; Mou, 2016). ‘one-line’ hybrid rice which could fix rice heterosis by means of apomictic reproduction and significantly reduce the cost of seed production, is the development direction of hybrid rice (Yuan, 2018).

Apomixis is an asexual propagation method through seeds in which the embryo is formed without the nuclear fusion of male and female gametes (Jia et al., 2015). In nature, apomixis and sexual reproduction could coexist in some apomictic species, such as *Hieracium*. when the sexual reproduction pathway fails, the expression of apomixis genes will be initiated, and the latter is an altered form of sexual reproduction (Catanach et al., 2006; Barcaccia and Albertini, 2013).

Apomictic pathway could be divided into either reduced gametophytic apomixis or diploid apomixis (Mu et al., 2001), the latter has been termed diplospory, apospory, and adventitious embryony (Koltunow, 1993; Mu et al., 2001; Conner et al., 2015). Somatic ovule cells that surround the embryo sac differentiate and have an embryogenic cell fate. With appropriate technical measures, somatic ovule cells of sexual plant might turn sexual reproduction into apomictic reproduction by aborting the egg cells and forming adventitious embryos.

Several genes, such as *BBM* (Boutilier et al., 2002; Anderson et al., 2017), *LEC* (Lotan et al., 1998; Kwong et al., 2003), and *WUS* (Mayer et al., 1998; Schoof et al., 2000) that could induce somatic embryogenesis in plants had been cloned in recent years. The *WUSCHEL* gene regulates the balance between stem cell proliferation and differentiation (Nardmann and Werr, 2006). Meanwhile, *WUS* is essential for regulating the growth of the stomium and septal cells during anther development, and for ovule development in *Arabidopsis* (Gros-Hardt et al., 2002; Deyhle et al., 2007). Upregulating *AtWUS* expression promotes somatic embryonic transition in Arabidopsis (Zuo et al., 2002), and improves regeneration of transgenic monocots (Gordon-Kamm et al., 2019), All these studies indicated that ectopically expressed *WUS* is able to transform tissues that are in a vegetative growth state into tissues with embryonic stem cell characteristics.

In this study, we first constructed one T-DNA containing three linked gene expression cassettes. The cassettes contained 1) the ribonuclease gene *Barnase* driven by the egg cell-specific promoter *AtDD45*, 2) the embryogenic gene *AtWUS* controlled by a somatic ovule cell-specific promoter, and 3) an inactivation gene *ZmAA1* driven by the pollen-specific promoter *pG47*. The second T-DNA carrying the *Barnase*-specific antagonist protein *Barstar* to inhibit the background expression of *Barnase* was constructed and then co-transformed with the first T-DNA into hybrid rice. We aimed to establish an engineering system by aborting the egg cells and forming adventitious embryos from somatic ovule cells such as the integument *via* expression of an embryogenic gene.

This study is a new exploration of a facultative apomictic reproductive system in hybrid rice. If this reproductive pathway transformation can occur in rice, it will provide a novel breakthrough for ‘one-line’ hybrid rice and will be valuable for fixing heterosis *via* synthetic apomixis in crops.

## Results

### Rationale of the experimental design

**Figure 1:**
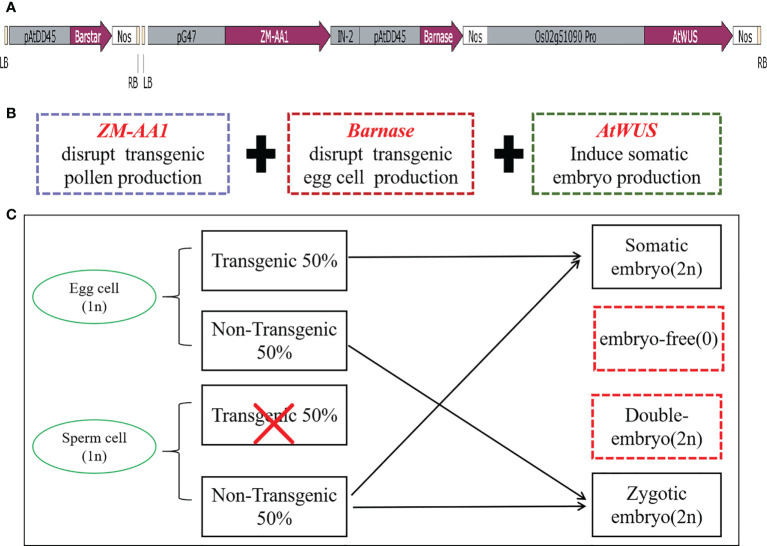
The transformation vector and model of the asexual seed propagation system. **(A)** Schematic diagram of the p22W vector construct containing two T-DNA regions. LB, left T-DNA border; pAtDD45, Arabidopsis DD45 promoter; Barstar, *Barstar* gene; NOS, nopaline synthase terminator; RB, right T-DNA border; pG47, pG47 promoter; ZM-AA1, *ZM-AA1* gene; IN-2, IN-2 terminator; Barnase, *Barnase* gene; Os02g51090pro, Os02g51090 promoter; AtWUS, Arabidopsis *WUS* gene. **(B)** The function of the three gene expression cassettes in the T-DNA. **(C)** Schematic model for generating asexual seed propagation in hybrid rice.

In order to induce somatic embryos and establish asexual reproduction, we constructed the p22W vector for *A. tumefaciens* with three expression cassettes in one T-DNA (**Figure 1A**): (1) the maize a-amylase gene *ZM-AA1* with the amyloplastic signal peptides under control of the pollen-specific *pG47* promoter to disrupt transgenic pollen production, which we have characterized previously (Xia et al., 2019); (2) the *Barnase* gene from *Bacillus amyloliquefaciens* that encodes a 12 kD small extracellular ribonuclease driven by the *Arabidopsis* egg cell-specific promoter *pDD45* to cause the death of egg cells (Steffen et al., 2007; Khanday et al., 2019); and (3) the *AtWUS* CDS under control of the rice ovule-specific promoter *Os02g51090* (Seagan and Lovett, 2014). We also introduced a *Barstar* expression cassette into the second T-DNA for transformation to inhibit the background expression of *Barnase* (**Figures 1A**, **B**). The *Barstar* gene encodes a *Barnase*-specific antagonist protein that is able to bind *Barnase*, rendering bacterially-expressed *Barnase* without enzymatic activity.

When the three cassettes are transformed into rice cells for designed genome engineering, the *ZM-AA1* cassette disrupts starch accumulation only in the transgenic pollen grains so that the transgenic pollen grains produced by the hemizygous transgenic plant are defective. In the absence of *Barstar*, the expression of *Barnase* usually causes host cell death, and ectopically expressed *AtWUS* will direct somatic cells into embryony, while the non-transgenic pollen grains are viable for pollination. The resulting transgenic plant produces male gametes (MG) of one genotype and female gametes (FG) of two genotypes. In the process of self-pollination, the nucellus cells form asexual embryos; egg cells, and sperm cells without the transgene form zygotic embryos, and the sperm cells without transgenes combine with the central cell to form endosperm, theoretically generating a 1:1 ratio of somatic embryos with transgenes and zygotic embryos without transgenes (**Figure 1C**).

### Ectopic expression of the *AtWUS* gene induced double-seedlings in transgenic hybrid rice

**Figure 2:**
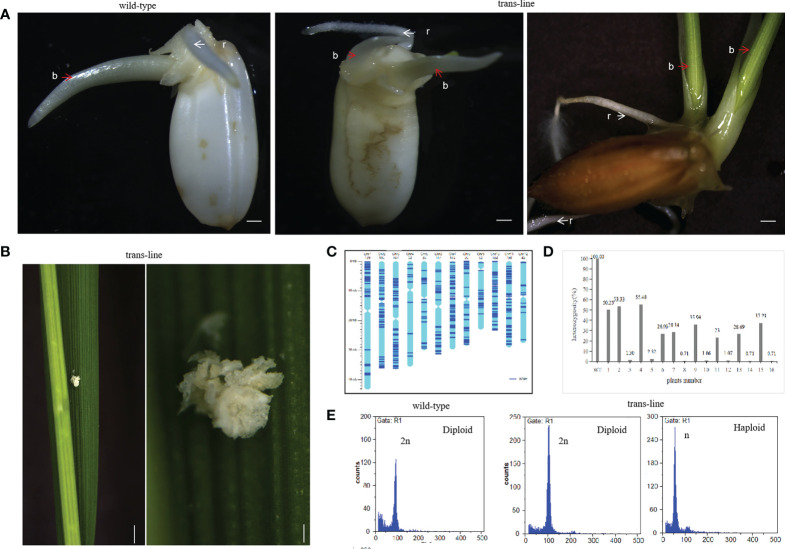
Recovery of double-seedlings in transgenic hybrid rice. **(A)** Seedlings in the wild-type (left; n=30) is normal with one bud (b; red arrow) and one root (r; white arrow). Double-seedlings in T_1_-generation (n=2; middle) and T_2_-generations (n=12; right) of could grown two buds(b; red arrow) and roots (r; white arrow) in trans-line. Scale bars, 1 mm. **(B)** Somatic embryo-like structures induced by *AtWUS* ectopic expression in trans-line leaves (n=20; left). Scale bars, 1 cm. The insets show magnified views of the somatic embryos (right). scale bars, 0.5 mm. **(C)** Determination of the genotype of wild-type 9Y using whole genome sequencing. The frame of each chromosome was constructed based on the *Oryza sativa* ssp. *japonica* genome version 6.0, with the scale on the left showing the length of the chromosome (Mb) and the names of the chromosomes shown across the top of the figure. The heterozygous SNP sites (AB) are marked in dark blue. **(D)** Genotyping analysis of trans-line. The plants are shown on the x-axis and the y-axis shows the heterozygosity. Wild-type 9Y (WT), F_1_ generation (1) and F_2_ generation progenies of 9Y (6) as control, double-seedlings from T_1_-generation (n=2; 2-5) and T_2_-generation (n=5; 7-16) were analysed. **(E)** Ploidy analysis of wild-type is diploid (n=3; left), one seedling of double-seedlings in trans-line is diploid (n=5; middle) and the other is haploid (n=5; right) by flow cytometry. The x-axis is the measure of relative fluorescence and the y-axis shows the number of nuclei.

The vector p22W was transformed into the rice hybrid 9Y using an *Agrobacterium*-mediated method. Seeds from transgenic line which contained *AtWUS* gene (hereafter trans-line) were obtained for germination. Some seeds could grow two buds and roots independently in trans-line, and were called double-seedlings (**Figure 2A**). The induction frequency of double-seedling was 0.11% to 0.13% in T_1_ generation and 0.21% to 0.45% in T_2_ generation. While, no double-seedlings were observed in wild-type 9Y (**Supplemental Table 1**). The single-seedling isolated from the double-seedlings also generate double-seedlings in subsequent generations. This showed that single-seedlings and double-seedlings contain the same genetic factors to develop into double-seedlings. The embryogenic calli structures were found on the third leaf onwards of adult seedlings (**Figure 2B**), these findings are similar to ectopic expression of *BBM1* in rice (Khanday et al., 2019).

PCR amplification of the *AtWUS* and *Barstar* genes was then carried out in trans-line plants. Among the 85 analyzed T_1_ progeny from trans-line, 28 of the seedlings were found to contain *AtWUS*, and *Barstar* gene. Among the 81 analyzed T_2_ progeny from different family, 22 of the seedlings were found to contain both the *AtWUS* and *Barstar* genes (**Supplemental Figure1A** and **Supplemental Table 1**). Double-seedlings were *AtWUS* and *Barstar* gene transformation positive from trans-line.

To demonstrate the genetic origin of the double-seedlings, we performed whole-genome sequencing on a diploid F_1_ wild-type 9Y plant, 22 T_1_ progeny plants including two double-seedlings, 20 single-seedlings, and a control untransformed F_2_ plant, 22 T_2_-generation progeny plants including five double-seedlings, ten single-seedlings, and an untransformed F_3_ control plant (Raw sequence data of these samples have been deposited in NCBI Short Read Archive with access number PRJNA870060). Analysis of sequence variants identified 1,302 SNPs in unique sequences distributed across the genome in the wild-type 9Y (**Figure 2C**). The 1,302 SNPs were determined to be heterozygous in control wild-type 9Y, and the heterozygosity of the wild-type was therefore set at 100%. The probability of F_2_ progeny from 9Y plant retaining heterozygosity by random segregation for just one SNP is P=50%, the heterozygosity of any single F_3_ progeny plant is P=25%. The maintenance of heterozygosity at all 1,302 SNPs on the different chromosomes by random segregation is P=(0.5)^1302 =^ 0. All of the progeny were not contain the heterozygous genotype as wild-type 9Y, which means that no seeds with somatic embryo were obtained. The heterozygosity of double-seedlings were 53.33% to 55.48%, and another was 1.30% to 2.32% in T_1_-generation. The heterozygosity of double-seedlings were from 23% to 37.23%, and another were 0.71 to 1.07% in T_2_-generation (**Figure 2D**). These data suggest that one of double-seedlings is heterozygous arises from the zygote after fertilization, and that another is homozygous. The ploidy levels of the above double-seedlings were confirmed by flow cytometry; one shoot is diploid and the other shoot is haploid (**Figure 2E**), which confirms that embryogenesis in double-seedlings arises from the zygote after fertilization and the other shoot is derived from an unfertilized gamete.

**Figure 3:**
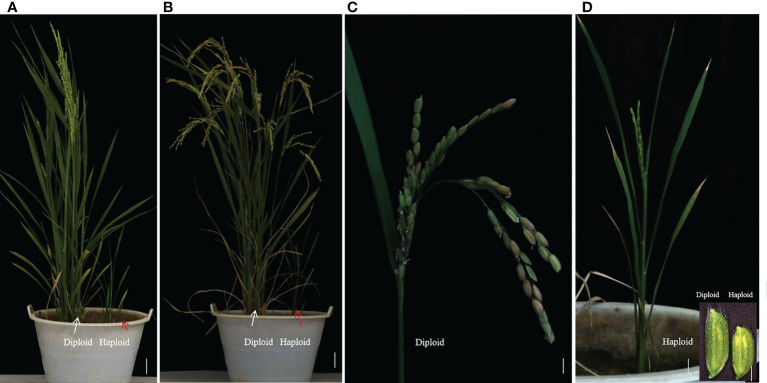
Characterization of double-seedlings in transgenic hybrid rice. **(A, B)** Two plants from double-seedlings are cultivated separately, Morphologies of the diploid (left; white arrow) and haploid (right; red arrow) is different at the heading stage **(A)** and at maturity. **(B)** Scale bars, 5 cm. **(C)** Panicle of the diploid in B is fertile. Scale bar, 2 cm. **(D)** panicle of haploid in B is sterile, and floret sizes of the diploid is larger than that of haploid plants. Scale bar, 2 cm. Inset scale bars, 2 mm.

Two plants from double-seedling were cultivated separately. Morphologies of the diploid and haploid were different at the heading stage and at maturity, haploids were more lower than diploid obviously (**Figures 3A, B**). We identified haploids by their small size compared with their diploid siblings as well as by their flowers. Due to defective meiosis, the haploids are sterile and the diploids were fertile with normal anther development (**Figures 3C, D**). Thus, ectopic expression of the *AtWUS* gene in the ovule is sufficient for the production of haploid plants. The diploids carry a non-identical set of alleles to the F_1_ mother plant, confirming that one of the double-seedlings (diploid) is generated by sexual reproduction and the other (haploid) is generated by asexual reproduction.

### Endosperm development is an autonomously programmed process in transgenic line

**Figure 4:**
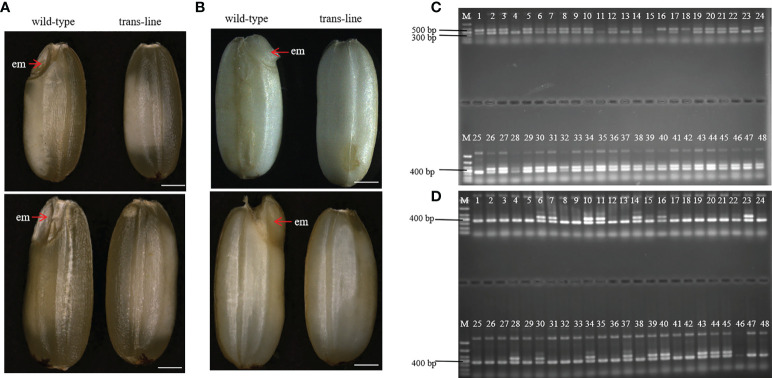
Identification and characterization of embryo-free seeds. **(A)** embryo-free seeds in trans-line and normal seeds in control wild-type are different in seed phenotype. Normal seeds with the embryo (em; red arrow) in control wild type (n=1000; left), but not in embryo-free seeds (n=59/1800; right). Scale bars, 1mm. **(B)** The embryo is germinated after the pre-germination treatment on normal seeds (left) and nothing changed in embryo-free seeds (right). Scale bars, 1 mm. **(C)** PCR detection of embryo-free seeds from trans-line, positive transgenic plantlets (lane 1) gave both a 456 bp fragment from *AtWUS* and a 364 bp fragment from the internal control *OSD1* gene. The 456 bp fragment from the *AtWUS* gene is not present in the control wild-type 9Y genome (lane 25), 91.3% percent of embryo-free seeds are transgene positive in trans-line (n=42/46; lanes 2-24, lanes 26-48). **(D)** Wild-type 9Y genome(lane 1, lane25) gave just a 364 bp fragment from the internal control *OSD1* gene. The 456 bp fragment from the *AtWUS* gene is amplified just at the percentage of 34.78% from normal seeds in trans-line (n=16/46; lanes 2-24, lanes 26-48).

During plant sexual reproduction, a double fertilization event initiates embryo and endosperm formation. The embryos are readily observed in normal seeds (**Figure 4A**). After pre-germination treatment, the embryos start to germinate, but embryo-free seeds only contain endosperm and do not germinate after pre-germination (**Figure 4B**). *Barnase* gene expression cassette causes the death of the egg cell, and self-pollination of trans-line results in embryo-free seeds. We obtained embryo-free seeds at frequencies of 2.6% to 3.8% due to single fertilization in T_1_ generation, and 0.75% to 3% in the T_2_ generation, no embryo-free seeds were observed in wild-type 9Y (**Supplemental Table 1**). This showed that contain the same genetic factors to develop into embryo-free seeds.

**Figure 5:**
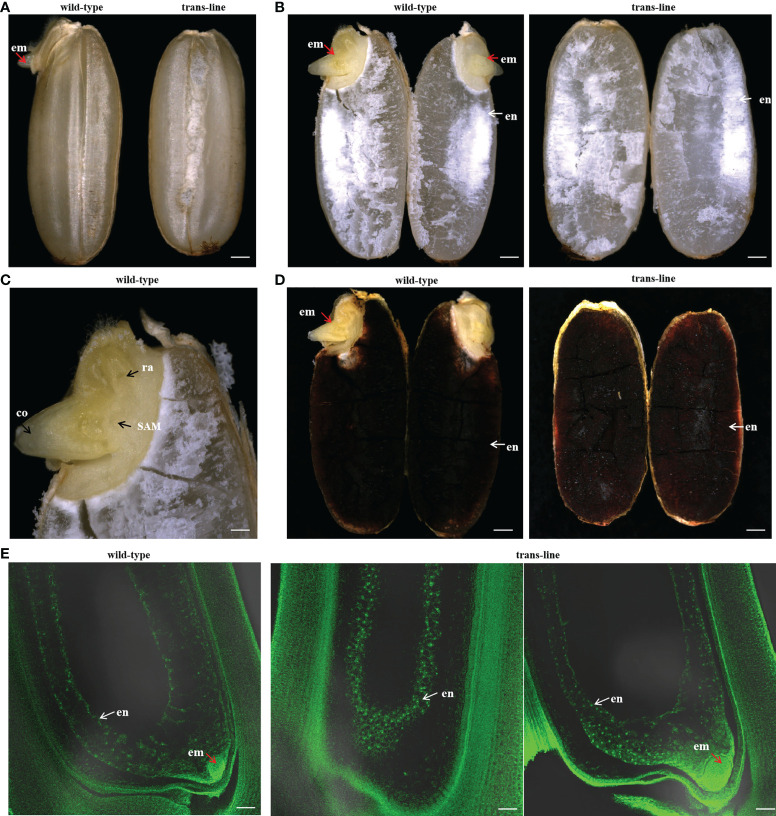
Development of embryo-free seeds in transgenic line. **(A)**Seeds were germinated normally with embryos (left; red arrow) one day after pre-germinated in wild-type 9Y (n=20), but embryo-free seeds without the germination of embryos formation (n=10/10; right) one day after pre-germinated in trans-line. Scale bars, 0.7 mm. **(B)** Embryo (left; em; red arrow) and endosperm (left; en; white arrow) are observed after a cross-section of seeds in wild-type (n=10; left), but just endosperm (right; white arrow) is observed with embryo-free seeds in trans-line (n=10; right). Scale bars, 0.7 mm. **(C)** Embryos show normal development in wild-type (n=10), co, coleoptiple; ra, radicle; SAM, shoot apical meristerm. Scale bars, 0.25 mm. **(D)** Endosperm turned into black when is staining with I_2_-KI in control wild-type (n=10; left) and trans-line(n=10; right). Scale bars, 0.7mm. **(E)** Development of embryo is not observed in carpels (n=1/20; middle) of the plant in trans-line at 3 days after pollination (DAP), or show normal development (n=19/20; right). In fertilized control wild-type carpels, the development of both embryo and endosperm in carpels (3DAPs) is observed (n=20; left). Scale bars, 35 µm.

Embryo and endosperm were observed in wild-type after a cross-section of the seeds, but just endosperm was observed with embryo-free seeds in trans-line (**Figures 5A, B**). Embryos showed normal development only in wild-type (**Figure 5C**). Endosperm turned into black when stained with I_2_-KI in wild-type and trans-line (**Figure 5D**). Development of embryo was not observed in carpels of the plant in trans-line at 3 days after pollination (DAP), or show normal development. In control wild-type, the development of both embryo and endosperm in carpels (3DAPs) were observed (**Figure 5E**).

We identified embryo-free seeds by PCR amplification of the *AtWUS* gene, the transgene positive rate for detecting the *AtWUS* gene in embryo-free seeds was 91.3%. But the transgene-positive rate was 34.78% in T_1_ progeny normal seeds from trans-line (**Figures 4C, D**). These results showed that embryo-free seeds were caused by transformation and carry the cassette: *Barnase* gene driven by the egg cell specific promoter *AtDD45*, which was lethal, so the egg cell was unable to form an embryo. However, the polar nuclei undergo fertilization to form normal endosperm in the embryo-free seeds. This showed that endosperm development is an autonomously programmed process that is independent of embryo formation.

## Discussion

Apomixis not only accelerates breeding procedures and the fixation of heterosis but also enables farmers to reduce investment and receive more benefits. From the developmental perspective, most natural apomixis is classified as facultative, because it involves both sexual and asexual modes of reproduction for the advancement of generations; hence, apomixis is widely thought to be a controlled or deviant form of sexual reproduction (Hand and Koltunow, 2014; Susmita et al., 2021; (Van Dijk et al., 1999). This theory suggested that if the sexual pathway is not initiated in an ovule, the apomixis pathway remains functional to form embryos. Apomictic reproduction requires the formation of embryos containing somatic chromosomes without fertilization and the development of endosperm either with or without fertilization.

In the case of adventitious embryony, non-zygotic embryos are formed outside the embryo sac and co-exist with sexual reproduction, because the sexual and asexual embryos share the nutritive endosperm produced from the sexual pathway for survival. Therefore, a seed containing multiple embryos is termed polyembryony (Hand and Koltunow, 2014).

In this study, double-seedlings were observed in transgenic line. one of double-seedlings was heterozygous diploid and the other seedling was homozygous haploid, which confirms that embryogenesis in double-seedlings arised from the zygote after fertilization and the other derived from an unfertilized gamete. *AtWUS* plays a key role in the occurrence of embryogenesis without fertilization. Thus, we guess other cells of the embryo sac such as synergids can develop into haploid embryo by expression of *AtWUS* gene driven by *Os02g51090* promoter, and it share the endosperm with the diploid embryo resulting from fertilization of egg cell and sperm cell. The plant of haploid is sterile and much smaller than that of diploid, when pre-germination in double-embryo seeds, haploid embryo can not compete with diploid embryo and death, this maybe why we observed double-seedlings at a low frequency.

In many other apomictic species, the development of functional endosperm takes place only when the pollen fertilizes the polar nuclei without egg cell fertilization (Bicknell and Koltunow, 2004). Rice is a sexually reproducing plant, and development of embryo-free seeds relies on the presence of haploid pollen that assures single fertilization and normal endosperm development. Embryo-free seeds obtained showed that in the absence of an embryo, endosperm can development normally as in the wild-type (**Figure 5**). These findings are similar to those previously reported in *Arabidopsis* (Xiong et al., 2021).

**Figure 6:**
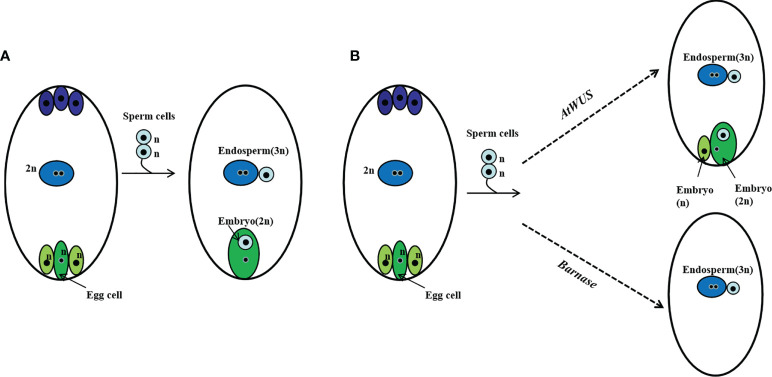
Schematic representation of polyembryony and embryo-free seed production. **(A)** Normal sexual reproduction and gamete fusion give rise to diploid progeny and triploid endosperm. **(B)** Ectopic expression of *AtWUS* induces other cells in the embryo sac to form embryos, the endosperm forms by fertilization of the central cell by a sperm cell. Asexual and sexual embryos share the endosperm formed during polyembryony. Ectopic expression of *Barnase* in the egg cell induces pollen single fertilization resulting in an embryo-free seed.

We suppose in this genetically regulated reproductive process, the egg cell develops into an embryo with fertilization and other cells of the embryo sac such as synergids develop into an embryo autonomously due to the ectopic expression of *AtWUS*, resulting in polyembryony. Because expression of *Barnase* in the egg cell causes a lethal egg cell and single fertilization occurs between the pollen and the polar nuclei without egg cell fertilization, resulting in embryo-free seeds (**Figure 6**).

The feasibility of fixing diploid clonal propagation of rice hybrids through seeds has been demonstrated by the MiMe strategy (Khanday et al., 2019; Wang et al., 2019). Hence, we are trying another engineering strategy for realizing apomictic reproduction of hybrid rice in the way of induce adventitious embryos. Though we did not obtained adventitious embryos in hybrid rice in this study, but the phenomenon of double-seedlings and embryo-free seeds in transgenic line were informative and strongly suggested that endosperm development is an autonomously organized process in rice. Nevertheless, the methods described here for asexual propagation shed new light on the use of agro-biotechnology for crop improvement. Expressing of *AtWUS* gene will have further application for haploid induction to rapidly obtain homozygous lines for crop breeding such as maize and wheat.

To engineer a completely asexual system involving autonomous endosperm formation is essential in a sexually reproducing crop. Whereas embryo formation may or may not require fertilization. Our findings will also provide further support for successful clonal propagation in synthetic apomixis.

## Materials and methods

### Plant materials and growth conditions

The hybrid rice (*Oryza sativa*) variety 9You 418 (9Y) is widely grown and is relatively easy to transform for a hybrid, so it was used as the transgenic acceptor in this study and as a wild-type (WT) control. The WT control was grown in a paddy field in the city of Changsha during the natural rice-growing seasons and maintained regularly. All lines created in this study were developed in the 9Y background. T_0_-, T_1_-, and T_2_-generation lines were grown in pots in the greenhouse.

### Plasmid construction and plant transformation

Left and right boundary sequences and multiple cloning sites were synthesized as oligonucleotides and ligated into pCAMBIA1300 (CAMBIA, Canberra, Australia) at the unique *Sac* II restriction enzyme site, generating the pC01 vector. The *Zm-AA1* expression cassette was excised as a single fragment by *Hind* III digestion from pFS4-11 (Xia et al., 2019), and was inserted into the pC01 vector that contained two T-DNA regions, generating pC02. The *Barstar* gene was inserted downstream of the *Arabidopsis DD45* promoter and upstream of the nopaline synthase terminator and cloned into the other T-DNA at the *Pac* I site, generating the vector pC03. The construct for egg-cell-specific expression of *Barnase* was made by cloning the *Barnase* gene between the *Arabidopsis DD45* promoter (Chen et al., 2012; Wu, 2012; Igawa et al., 2013) and the nopaline synthase terminator. The ovule-specific expression of *AtWUS* (AT2G17950) was engineered by cloning the full-length coding sequence (CDS) of *AtWUS* downstream of the rice ovule-specific *Os02g51090* promoter and upstream of the nopaline synthase terminator (Li et al., 2004; Seagan and Lovett, 2014). The two fragments were synthesized by GenScript and then cloned into pC03 downstream of *Zm-AA1* at the *Asc* I site to make p22W.

The p22W construct was introduced into *Agrobacterium tumefaciens* EHA105 and transformed into hybrid rice 9Y. Callus induction and plant regeneration were performed as previously described (Xia et al., 2019). Transgenic callus selected on medium containing the antibiotic hygromycin.

### Pre-germination

T_1_- and T_2_-generation seeds were randomly sampled in a paper bag, and soaked in a covered container containing purified water at 37°C for 24 hours. The paper bag was removed, and the seeds were wreathed by a wet towel and allowed to sprout in the greenhouse at a temperature of 28°C for 24-48 hours. The seeds were then removed to a dish containing filter paper, and seed germination was observed for seven consecutive days.

### Genomic DNA extraction and transgene determination

PCR amplification screens were used to confirm the presence of the *AtWUS* and *Barstar* genes in the transgenic plants. Total genomic DNA was extracted from leaf tissue of both transformed and untransformed plants using the CTAB pyrolysis method (Long et al., 2018). Total genomic DNA was extracted from seeds and endosperm tissue by the Inspection and Testing Center of the State Key Laboratory of Hybrid Rice in Changsha, Hunan Province, China. The PCR assays were performed using 2×Taq PCR Mix (Tiangen). The *AtWUS* CDS sequence was amplified with the gene-specific primers *WUS*-F (5’-CTGAGACAGTTCGGAAAGATTGA-3’) and *WUS*-R (5’-GAAGTTGTAAGGTGCAGATGAGTAA-3’). The rice *OSD1* gene (*Os02g37850*) was used as the internal control for normalization of gene expression. The *Barstar* gene sequence was amplified with the specific primers Bar-3F (5’-AATCCTTTCCCATTCCTCCCACT-3’) and Bar-3R (5’-GCTTGCTTTGTTCAAACTGCCTC-3’).

### Imaging and microscopy

Plants and organs were photographed with a Cannon EOS5D MarkIII digital camera. Double-embryos, embryo-free seeds, and embryo-like structures were observed and imaged with a Leica MZ16FA microscope and a Zeiss SmartZoom5 microscope, respectively. Embryo-free seeds were analysised by I_2_-KI staining after cross-section and imaged with a Zeiss SmartZoom5 microscope.

### Cytological observation

For phenotypic analysis of embryo-free seeds, self-pollinated flowers from trans-line were collected at 3 DAP. carpels fixed in FAA solution for 24 h at room temperature and then stored in 70% ethanol. Prepare carpels using whole stain-clearing technique. The carpels were scanned with a confocal laser microscope (Zeiss LSM880).

### Genotyping and statistical analysis

Genotyping by pinpoint sequencing of multiplex PCR products (Higentec, Changsha and GENOSEQ, Wuhan). We designed characteristic primers for the 1,048 target regions based on the rice genome sequence, and the specific primers were used to amplify 1,048 target regions at the same time by Multiplex-PCR (150-280 bp/per region). We then prepared libraries for next-generation sequencing of the PCR amplification products. Thus, we were able to obtain the genotypes for 5,400 SNP markers within the 1,048 target regions. Genotype calling was performed in the whole genome region using these SNPs which were heterozygous in the parent. The final list of 1,302 high-quality heterozygous SNPs were analyzed for segregation in the progeny plants and the recombination map was constructed for each chromosome (Higentec, Changsha).

### Flow cytometry

Leaf cell ploidy was determined by estimating nuclear DNA content using flow cytometry (Ploidy Expert, Beijing). Nuclei for fluorescence-activated cell sorting analysis and DNA-content estimation were prepared using a method described previously (Wang et al., 2019).

## Data availability statement

The original contributions presented in the study are publicly available. This data can be found here: NCBI, PRJNA870060.

## Author contributions

MC and YX conceived and supervised the project. MC and YX designed the experiments. YX, YW, YH, YZ, JD, NT and JT performed the experiments. YX and YH wrote the manuscript with the help of MC. All authors read and approved the final manuscript.

## Funding

This research was financially supported by the Natural Science Foundation of Hainan province (321MS108), the Natural Science Foundation of Changsha city(kq2202348), the Hainan Major Science and Technology Projects(ZDKJ202001) and the 2020 Research Program of Sanya Yazhou Bay Science and Technology City (202002006).

## Conflict of interest

The authors declare that the research was conducted in the absence of any commercial or financial relationships that could be construed as a potential conflict of interest.

## Publisher’s note

All claims expressed in this article are solely those of the authors and do not necessarily represent those of their affiliated organizations, or those of the publisher, the editors and the reviewers. Any product that may be evaluated in this article, or claim that may be made by its manufacturer, is not guaranteed or endorsed by the publisher.

## Supplementary material

Supplementary Figure 1 PCR analysis of the transgene in particial progeny plants. **(A)** PCR analysis of the *AtWUS* gene in T_1_-generation of trans-line. T_0_ transgenic plantlets (lane 1), the wild type 9Y genome (lane 2), T_1_ progeny plants(lanes 3-24), positive transgene gave both a 456 bp fragment from *AtWUS* and a 364 bp fragment from the internal control *OSD1* gene. The wild type 9Y genome just give a 364 bp fragment from the internal control *OSD1* gene. **(B)** PCR detection of the *Barstar* gene in T_1_ plants of trans-line, T_0_ transgenic plantlets (lane 1), The wild type 9Y genome (lane 2), T_1_ progeny plants(lanes 3-24), positive transgene gave just a 345 bp fragment. **(C)** PCR analysis of the *AtWUS* gene in T_2_-generation of trans-line. The wild type 9Y genome (lane 1), T_2_ progeny plants (lanes 3-14). **(D)** PCR analysis of the *Barstar* gene in T_2_-generation of trans-line. The wild type 9Y genome (lane 1), T_2_ progeny plants(lanes 3-14).

Supplementary Table 1 Double-seedings and embryo-free seeds induction in transgenic rice.

Supplementary Table 2 Detecting the exogenous gene in transgenic rice.
