# Supplementary figures and images for: Double-seedlings and embryo-free seeds generated by genetic engineering
Source: Front Plant Sci. 2022 Oct 3;13:999031. doi: 10.3389/fpls.2022.999031 (PMC9576183; doi:10.3389/fpls.2022.999031)

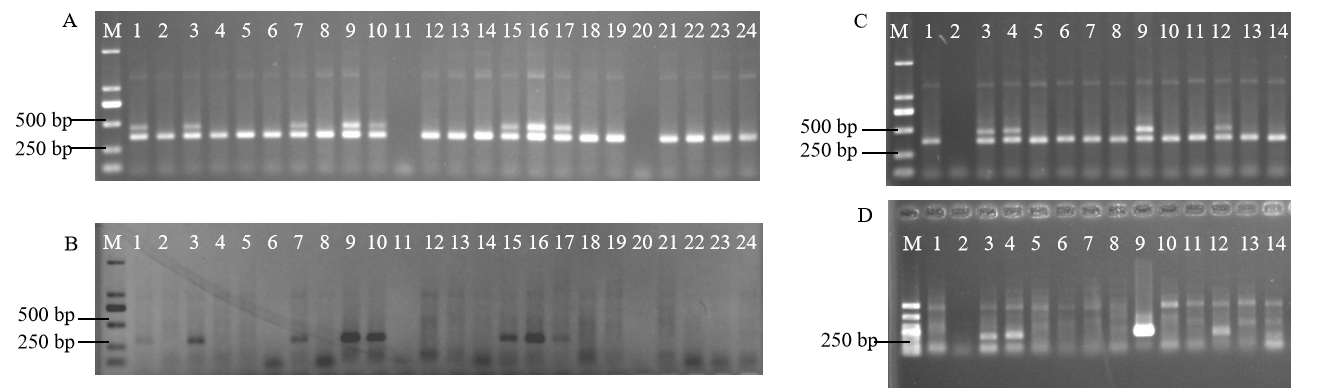

Supplement: Supplementary Figure 1 — PCR analysis of the transgene in particial progeny plants. (A) PCR analysis of the AtWUS gene in T1-generation of trans-line. T0 transgenic plantlets (lane 1), the wild type 9Y genome (lane 2), T1 progeny plants(lanes 3-24), positive transgene gave both a 456 bp fragment from AtWUS and a 364 bp fragment from the internal control OSD1 gene. The wild type 9Y genome just give a 364 bp fragment from the internal control OSD1 gene. (B) PCR detection of the Barstar gene in T1 plants of trans-line, T0 transgenic plantlets (lane 1), The wild type 9Y genome (lane 2), T1 progeny plants(lanes 3-24), positive transgene gave just a 345 bp fragment. (C) PCR analysis of the AtWUS gene in T2-generation of trans-line. The wild type 9Y genome (lane 1), T2 progeny plants (lanes 3-14). (D) PCR analysis of the Barstar gene in T2-generation of trans-line. The wild type 9Y genome (lane 1), T2 progeny plants(lanes 3-14). [file Image_1.tif]
