# Supplementary material for: Double-seedlings and embryo-free seeds generated by genetic engineering
Source: Front Plant Sci. 2022 Oct 3;13:999031. doi: 10.3389/fpls.2022.999031 (PMC9576183; doi:10.3389/fpls.2022.999031)
Supplement: Supplementary Table 1 — Double-seedings and embryo-free seeds induction in transgenic rice. [file Table_1.doc]

**Supplemental Table 1 Double-seedings and embryo-free seeds induction in transgenic rice**

| generation | Lines | seeds tested | single -seedlings | Double-seedlings(%) | embryo-free seeds (%) |
| --- | --- | --- | --- | --- | --- |
|  | 9Y | 1000 | 979 | 0 (0.00%) | 0 (0.00%) |
| T_1_ | #1 | 1000 | 850 | 1 (0.11%) | 38 (3.80%) |
|  | #4 | 800 | 735 | 1 (0.13%) | 21 (2.63%) |
|  | #1-1 | 600 | 502 | 2 (0.39%) | 9 (1.50%) |
| T_2_ | #1-22 | 600 | 466 | 1 (0.21%) | 7 (1.17%) |
|  | #1-47 | 800 | 642 | 2 (0.31%) | 6 (0.75%) |
|  | #4-1 | 700 | 598 | 2 (0.32%) | 21 (3.00%) |
|  | #4-18 | 700 | 567 | 2 (0.35%) | 9 (1.29%) |
|  | #4-41 | 800 | 653 | 3 (0.45%) | 9 (1.13%) |
