# Supplementary material for: Double-seedlings and embryo-free seeds generated by genetic engineering
Source: Front Plant Sci. 2022 Oct 3;13:999031. doi: 10.3389/fpls.2022.999031 (PMC9576183; doi:10.3389/fpls.2022.999031)
Supplement: Supplementary Table 2 — Detecting the exogenous gene in transgenic rice. [file Table_2.doc]

**Supplemental Table 2 Detecting the exogenous gene in transgenic rice**

| generation | Lines | plants tested | *WUS* positive | *Barstar* positive | positive transformation rate(%) |
| --- | --- | --- | --- | --- | --- |
| #1 | T_1_ | 36 | 12 | 12 | 33.33 |
|  | T_2_ | 40 | 11 | 11 | 27.50 |
| #4 | T_1_ | 49 | 17 | 17 | 34.69 |
|  | T_2_ | 41 | 11 | 11 | 26.83 |
